# Supplementary material for: Microbial community and geochemical analyses of trans-trench sediments for understanding the roles of hadal environments
Source: ISME J. 2019 Dec 11;14(3):740–56. doi: 10.1038/s41396-019-0564-z (PMC7031335; doi:10.1038/s41396-019-0564-z)
Supplement: Supplementary file 1 — Supplementary Information [file 41396_2019_564_MOESM1_ESM.docx]

**Supplementary Information**

**Microbial community and geochemical analyses of trans-trench sediments for understanding the roles of hadal environments**

Satoshi Hiraoka^1*^, Miho Hirai^2^, Yohei Matsui^3,4^, Akiko Makabe^2^, Hiroaki Minegishi^2^, Miwako Tsuda^2^, Juliarni^3^, Eugenio Rastelli^5^, Roberto Danovaro^5,6^, Cinzia Corinaldesi^7^, Tomo Kitahashi^8^, Eiji Tasumi^2^, Manabu Nishizawa^2^, Ken Takai^2^, Hidetaka Nomaki^2^, and Takuro Nunoura^1*^

*Correspondence should be addressed to:

hiraokas@jamstec.go.jp

takuron@jamstec.go.jp

This PDF file includes:

- Figure S1 to S12.
- Table S1 to S3.
- Overview of Supplementary Data 1 to 3

**Supplementary Figures**

**
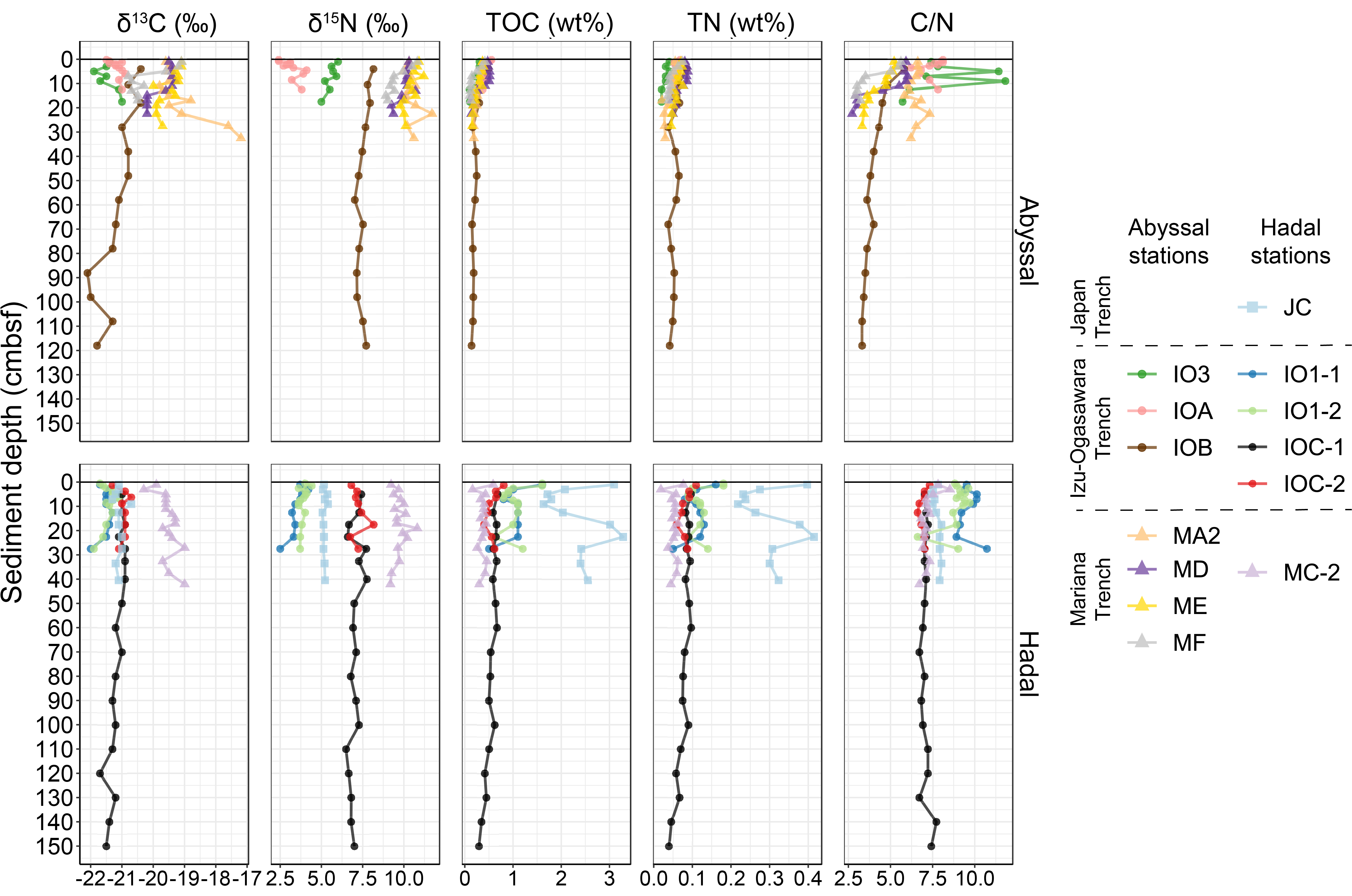
**

**Figure S1.** Concentrations of C and N isotopes (δ^13^C and δ^15^N, respectively), total organic carbon (TOC), total nitrogen (TN), and C/N ratio of the surface sediments in abyssal (upper panels) and hadal (lower panels) stations.


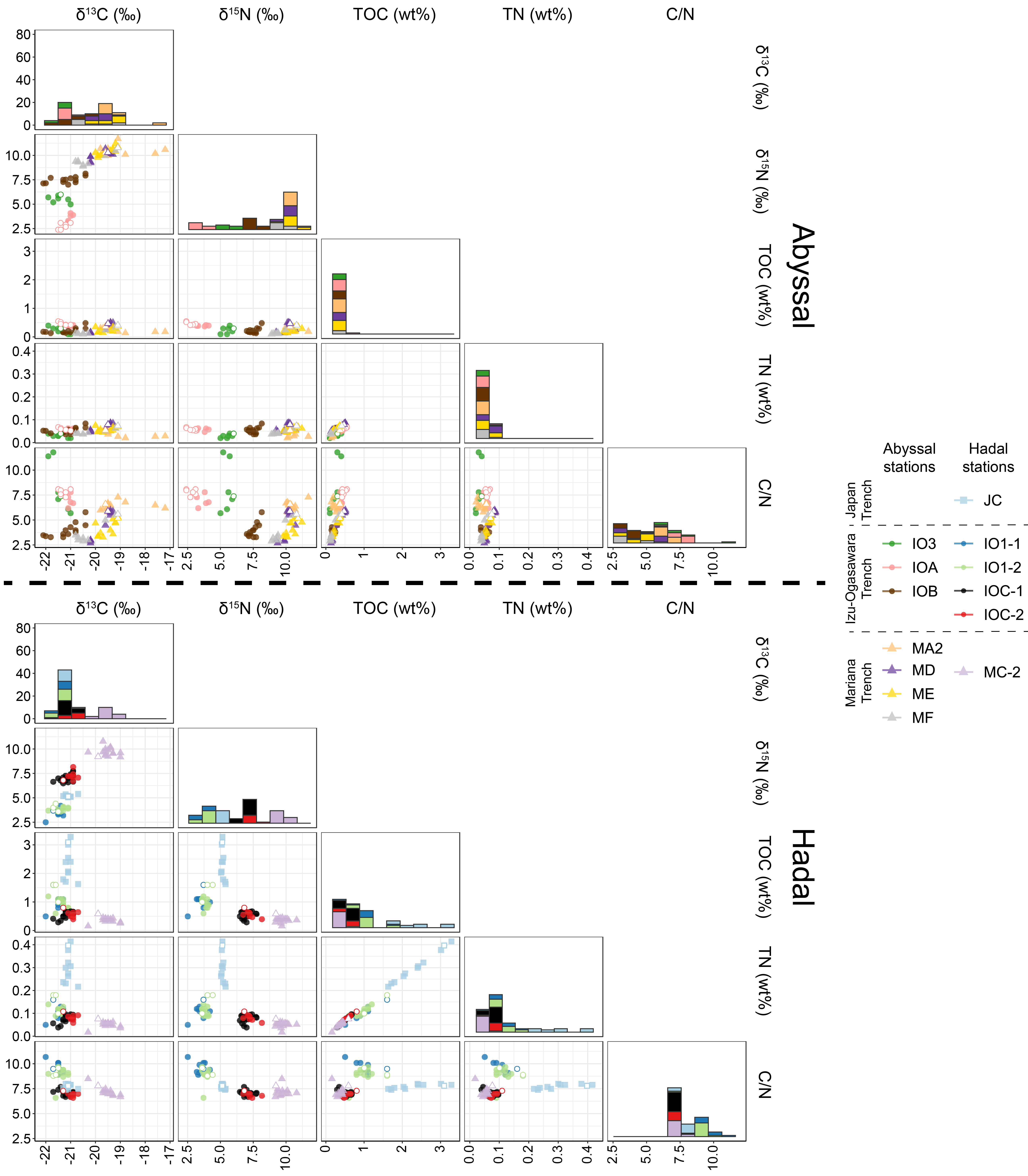


**Figure S2.** Pairwise relations of total organic carbon (TOC), total nitrogen (TN), C and N isotopes (δ^13^C and δ^15^N, respectively), and C/N ratio of the sediments in abyssal (upper panels) and hadal (lower panels) stations. The lower triangle panels indicate scatter plots of each pair of the geochemical measurements. Each sediment core is coded by color. Blank nodes represent measurements from surface layer (<3 cmbsf). The diagonal plots indicate cumulative histograms of measurements.


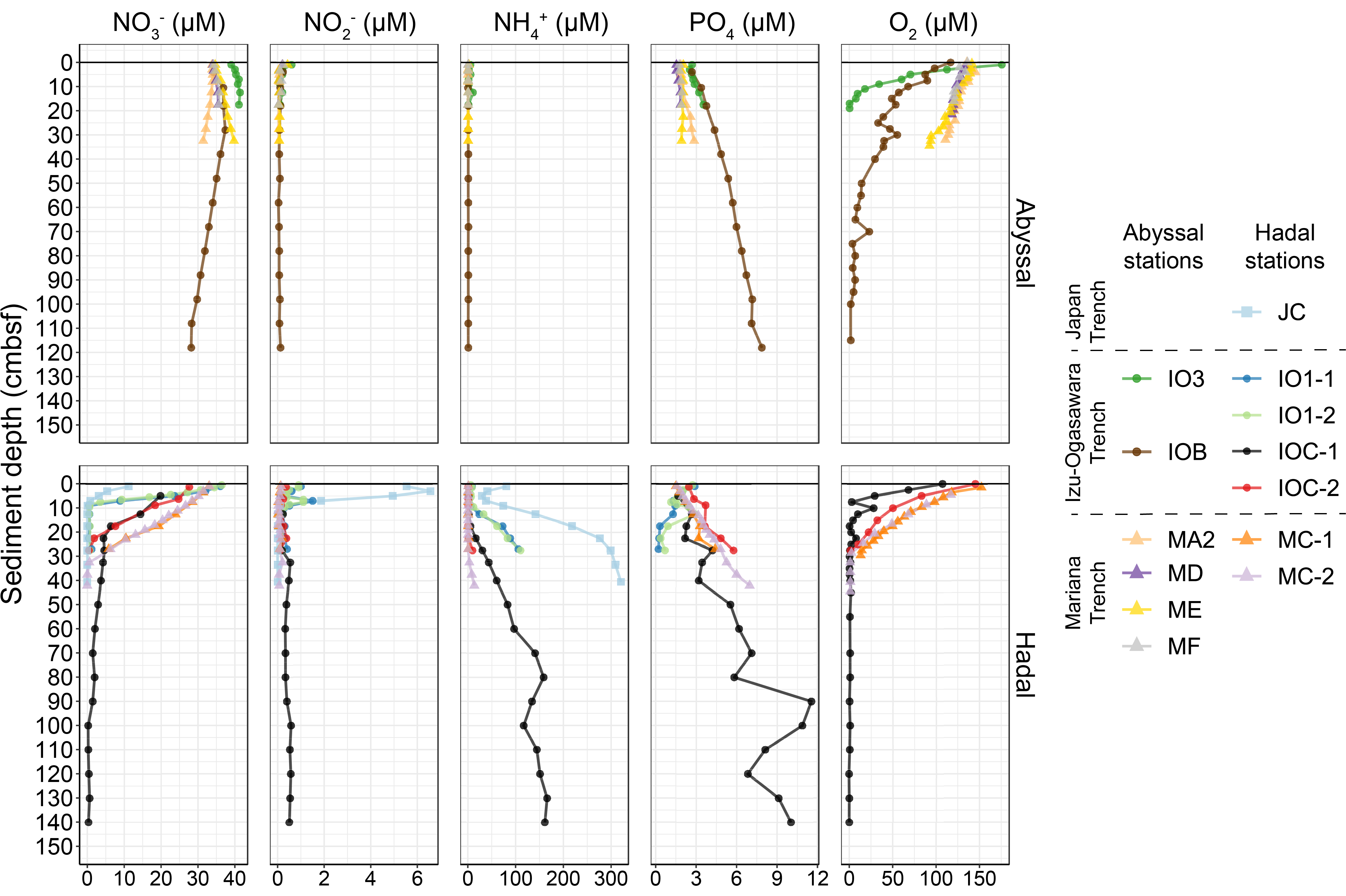


**Figure S3.** Porewater chemistry of the surface sediments in abyssal (upper panels) and hadal (lower panels) stations.


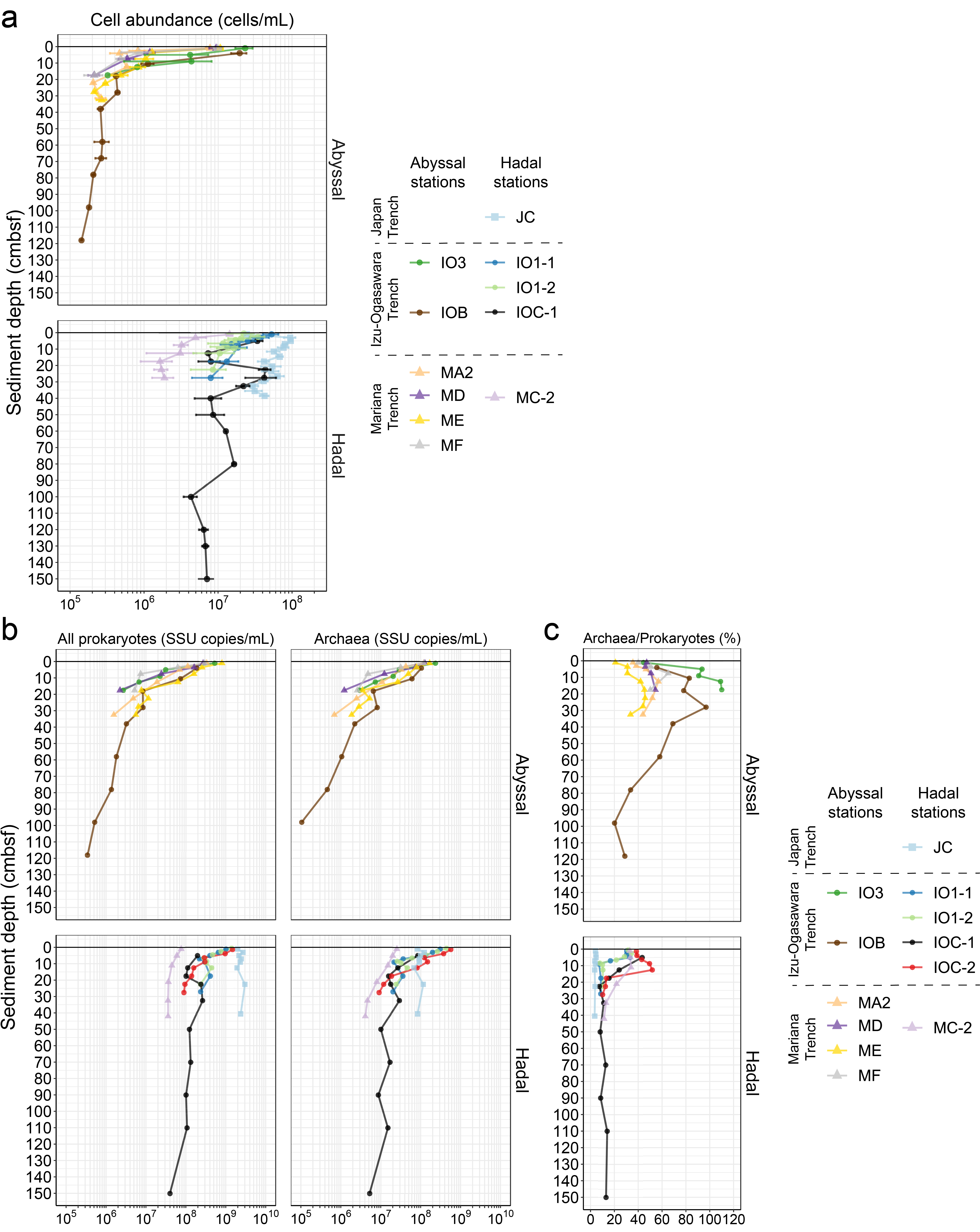


**Figure S4.** The abundance of microbes and ratios of archaea in each sediment core from abyssal (upper panels) and hadal (lower panels) stations. The abundances were measured using (**a**) cell counting and (**b**) qPCR techniques. The X-axes represent cell counts and SSU rRNA gene copies per milliliter of sediment, respectively. The error bars represent standard deviation. (**c**) Ratios of archaea/prokaryotes were calculated using qPCR data.


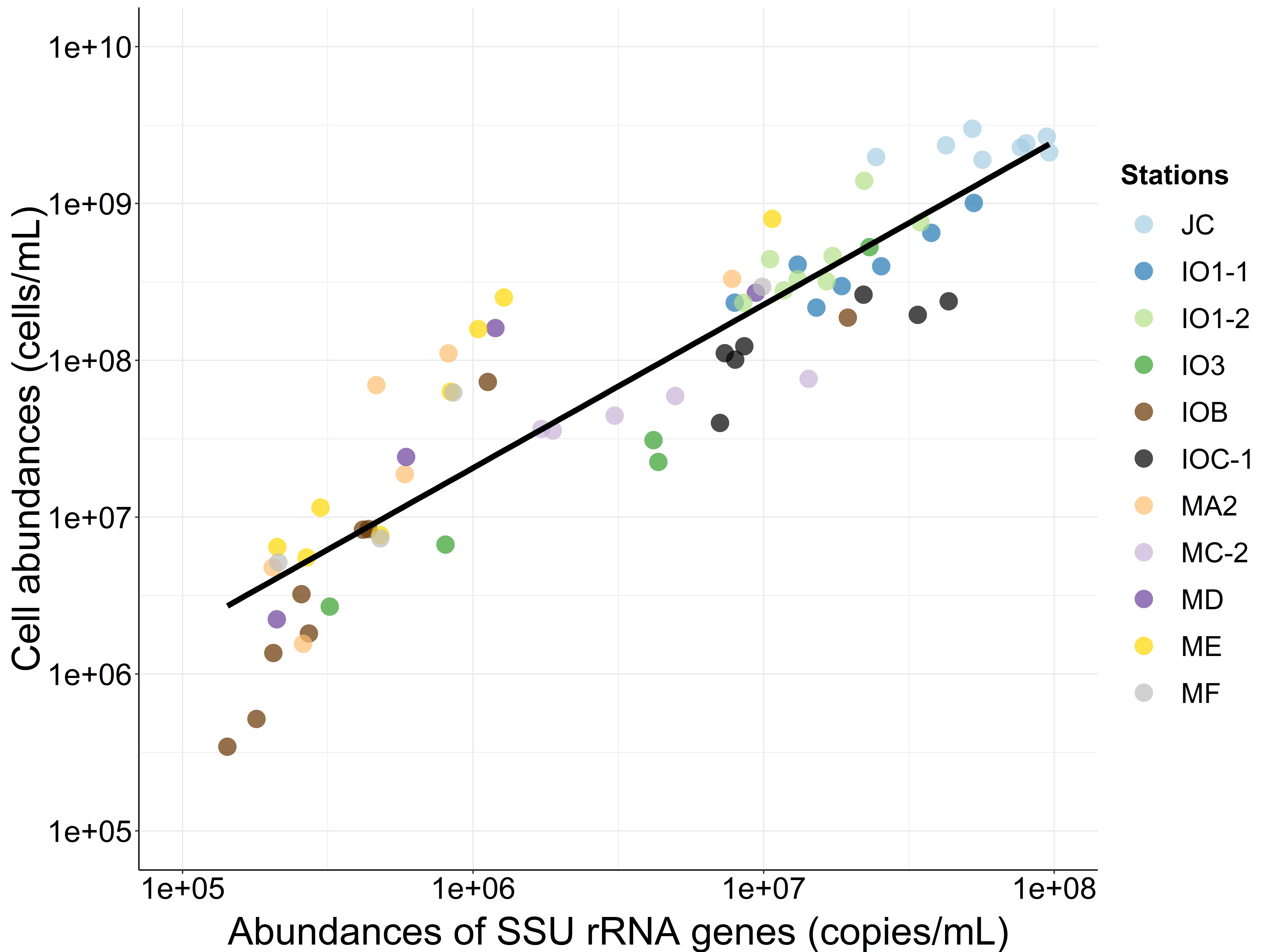


**Figure S5.** Relationship between qPCR counts and cell counts. Only sediment samples in which both cell counts and qPCR were conducted were included in this analysis. The equation of the linear regression line was y = 28.2x + 4.6e+06 (r^2^=0.75). Each sediment core is coded by color.


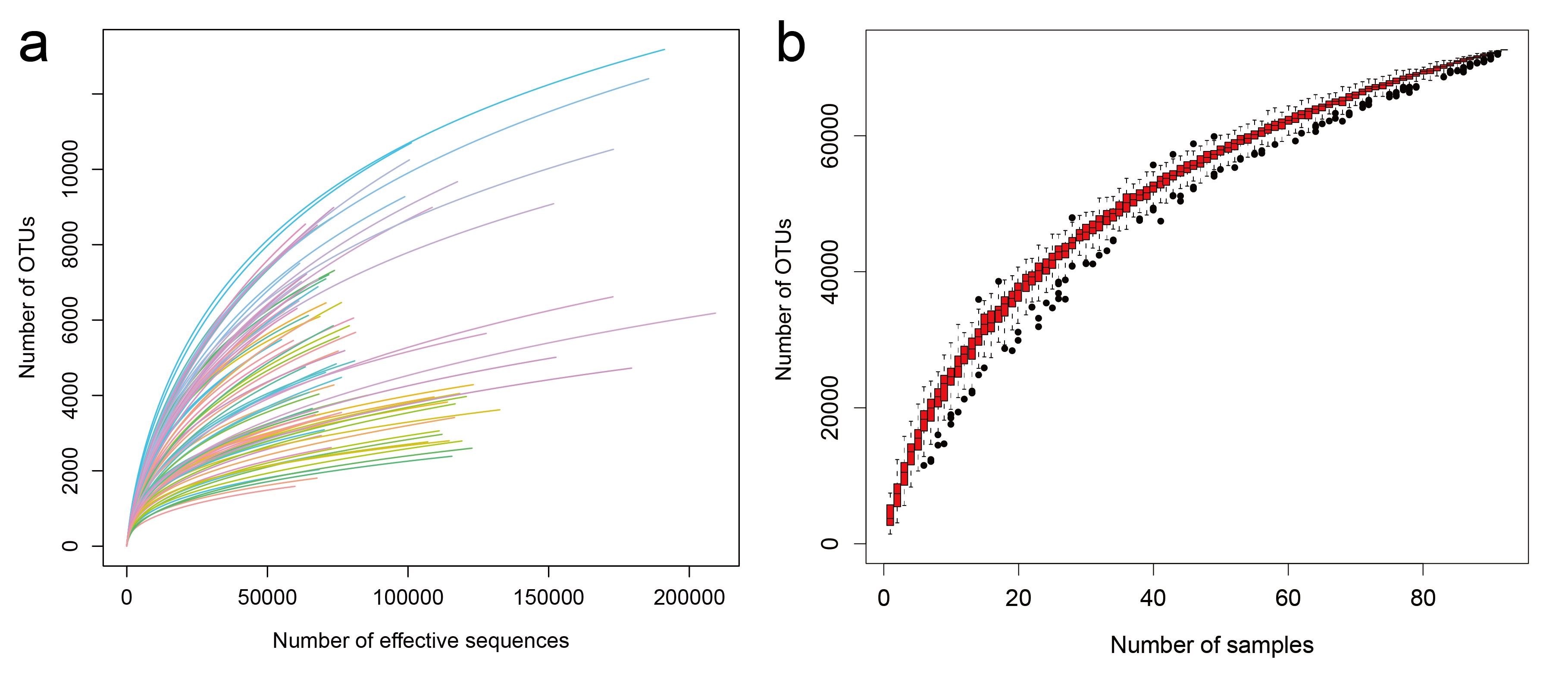


**Figure S6.** (a) Rarefaction curves of OTU numbers against each of examined samples. (b) Collectors curve of OTU numbers against the number of examined samples.


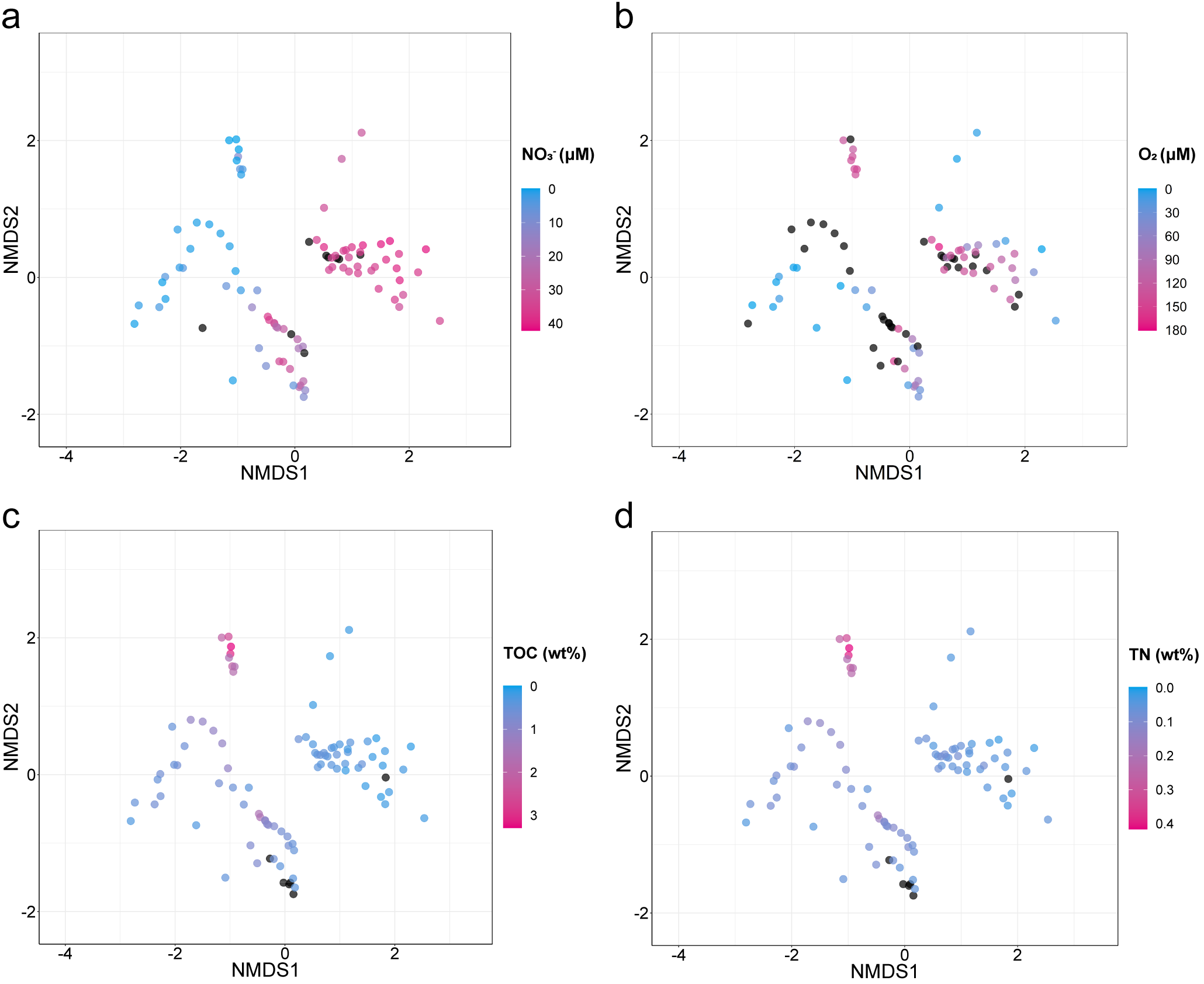


**Figure S7.** Nonmetric multidimensional scaling (NMDS) plots for OTU compositions. The sediment samples were colored depending on (**a**) NO_3_^-^, (**b**) O_2_, (**c**) TOC, and (**d**) TN concentrations. Samples without corresponding geochemical data were colored in black.

**
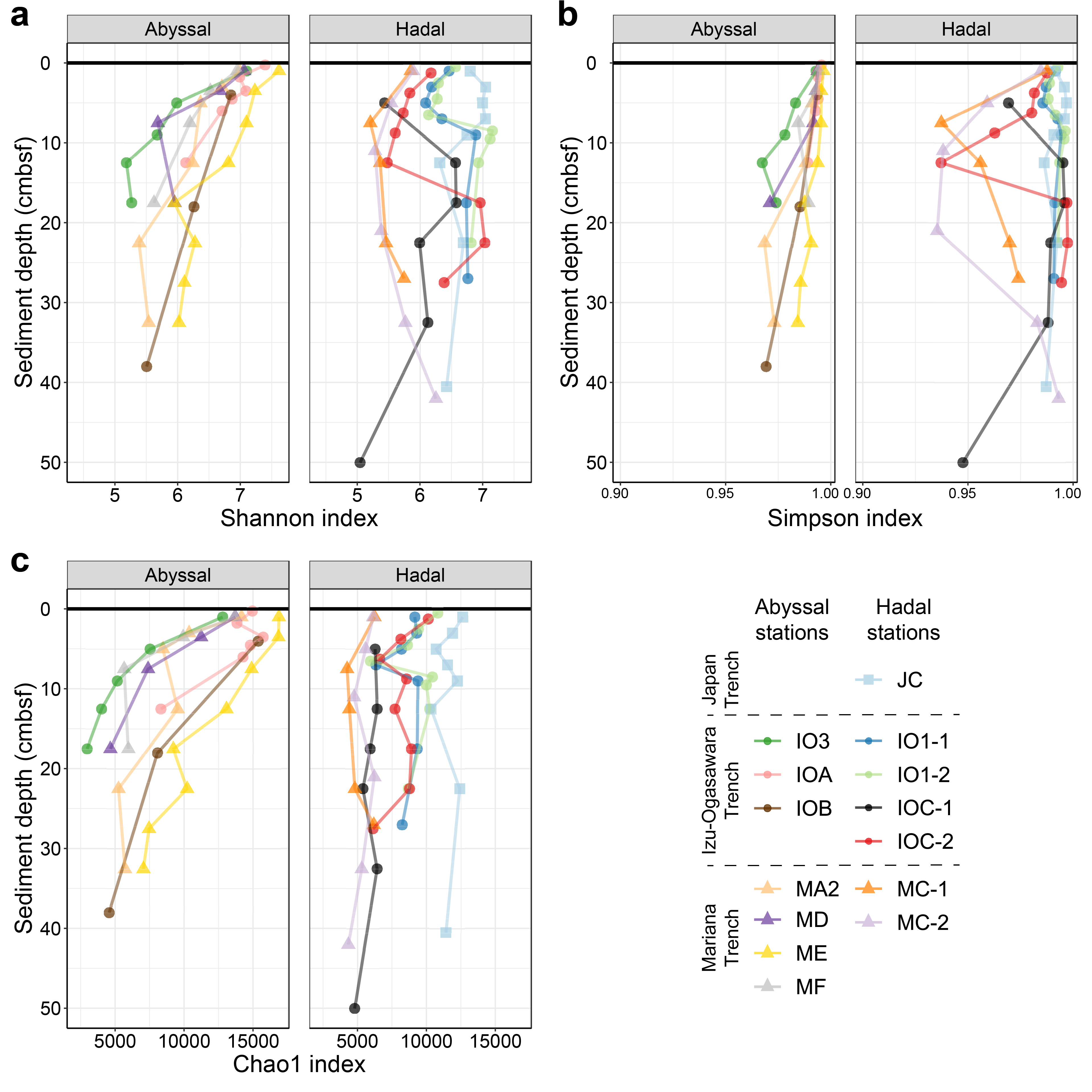
**

**Figure S8.** (**a**) Shannon diversity index, (**b**) Simpson diversity index, and (**c**) Chao1 estimated richness at the OTU level versus sediment depth from abyssal (left panel) and hadal (right panel) stations. The colored lines represent the values of each sampling station. Data from layers ranged between 0 to 50 cmbsf are shown in this figure.


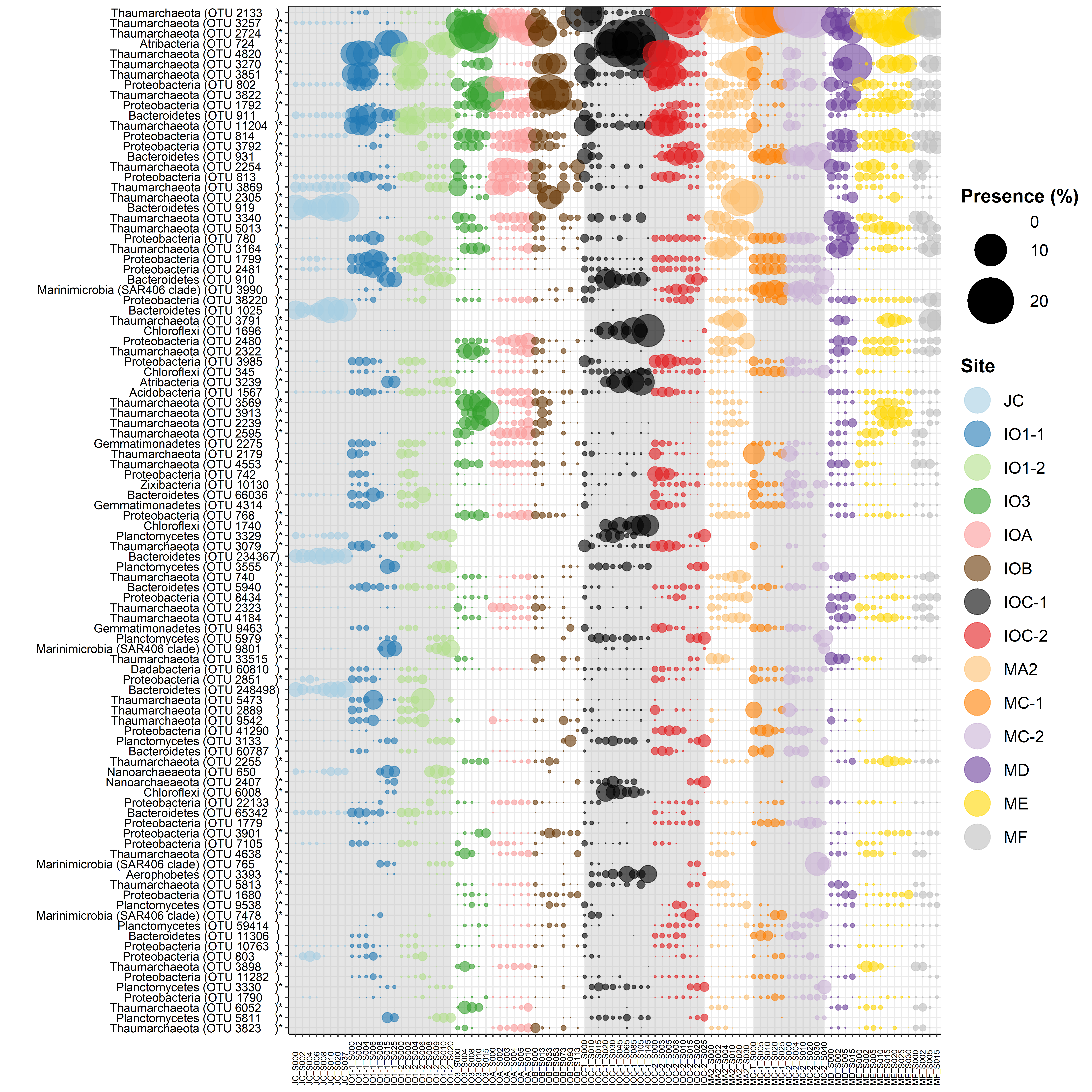


**Figure S9.** Comparative profiles of the top 100 most abundant OTUs in the sequencing pool. The OTUs were sorted in descending order. Bubbles are colored by sampling station and bubble sizes correspond to relative abundances. The white and gray backgrounds represent abyssal and hadal sediment samples, respectively. OTUs with asterisks indicate statistical significance of localization in either the abyssal or hadal stations (p<0.05, U-test, Bonferroni correction).


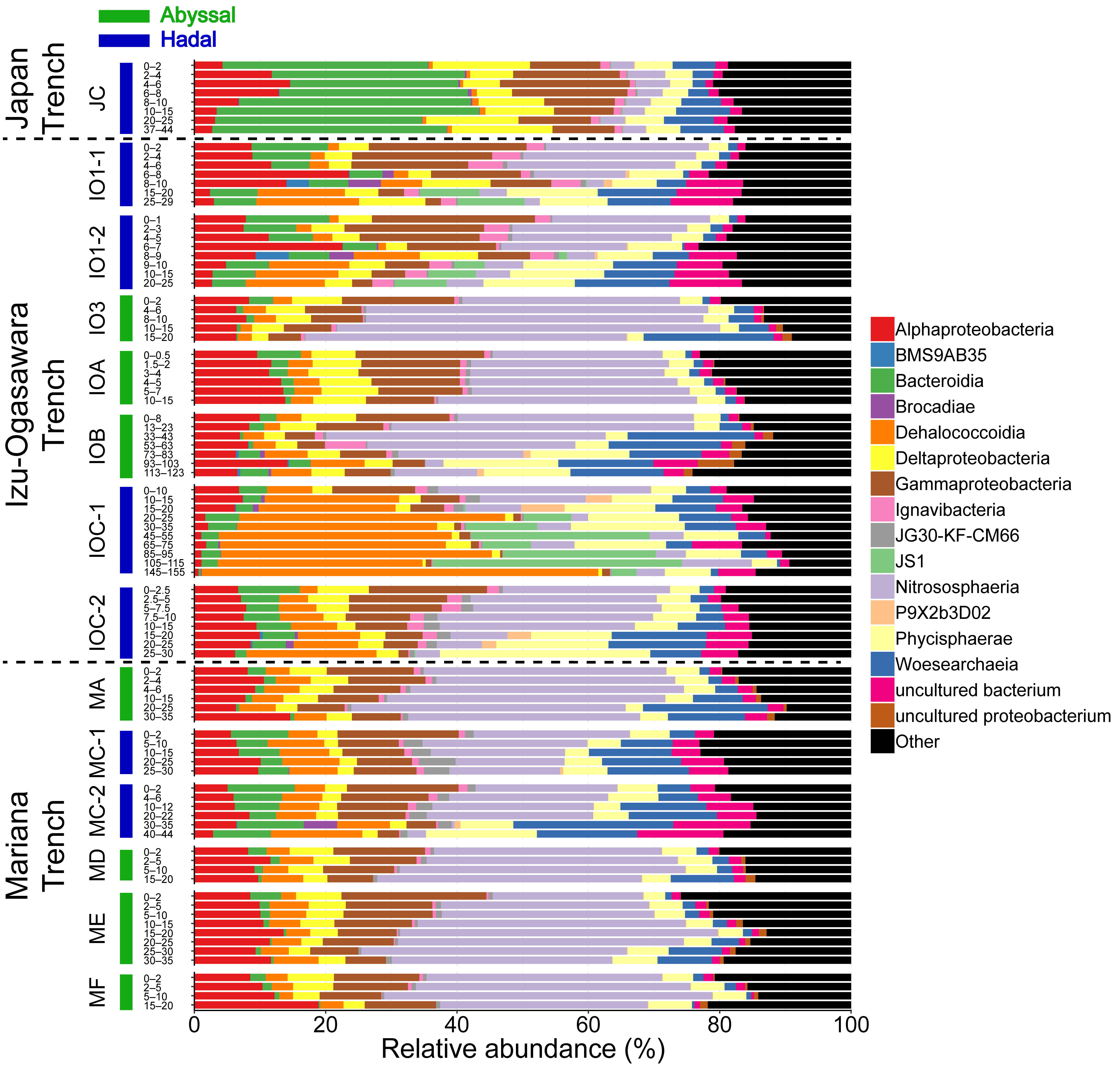


**Figure S10.** Relative abundances of sequences at the class level. Groups demonstrating <5% abundance are summarized as ‘Other’. Sediment samples retrieved from deep sediment layers (>s50 cmbsf) are indicated by surrounding black rectangles.


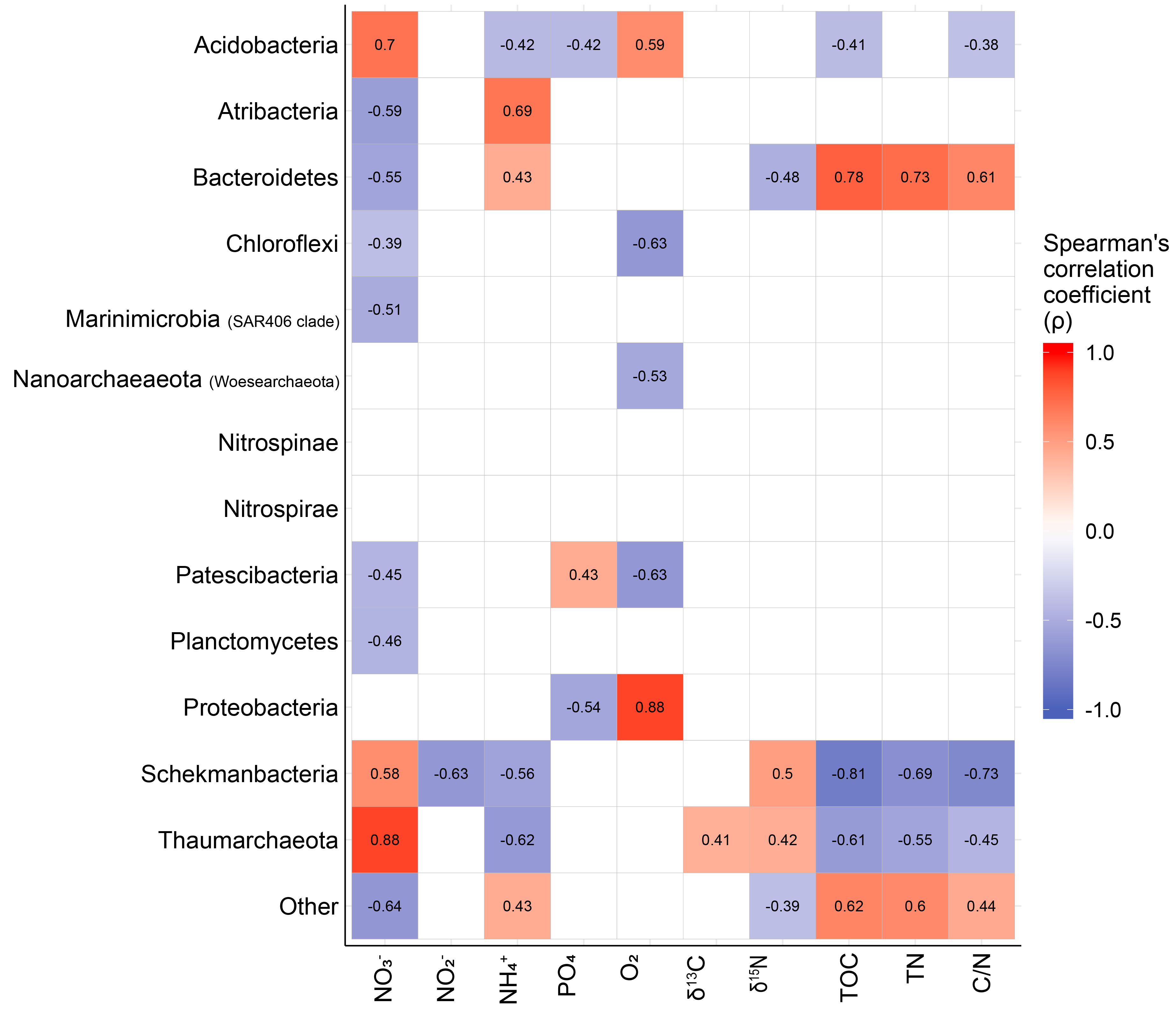


**Figure S11.** A Spearman’s correlation matrix between geochemical variables and taxa at phylum level. Data with p < 0.05 after Bonferroni correction were shown. Color with red shows positive correlation while blue shows negative correlation. Numbers of sediment sample used for calculation of correlation coefficient were as follow: N=89 (NO_3_^-^, NO_2_^-^, NH_4_^+^), N=81 (PO_4_), N=65 (O^­^_2_), and N=86 (δ^13^C, δ^15^N, TOC, TN, C/N).


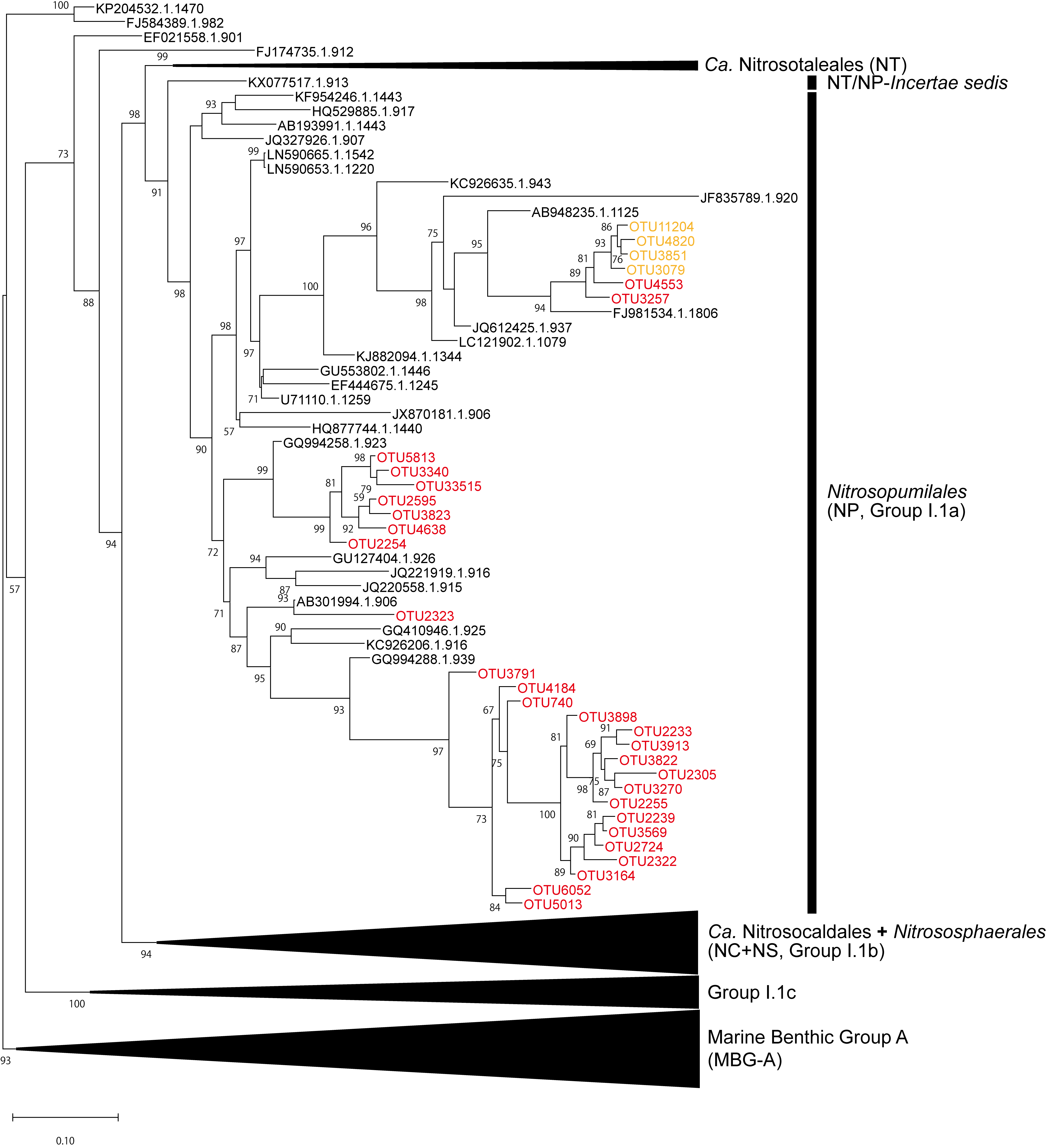


**Figure S12.** Phylogenetic tree of Thaumarchaeota SSU rRNA gene sequences. Nodes colored by red and orange represent OTUs belonging to the co-occurrence group A and D, respectively. Numbers adjusted with edges indicate bootstrap supports, and only values >50 are shown. Scale bar represents the estimated number of substitutions per site.

**Supplementary Tables**

**Table S1.** Station descriptions and sequencing statistics of each sediment sample

**Table S2.** Primers, probes, and amplification conditions of qPCR analyses

**Table S3.** Primers and adapters used for SSU rRNA gene PCR amplification

**Supplementary Data**

**Data 1.** Relative abundances of the top 100 most abundant OTUs in the sequencing pool.

**Data 2.** Summarized geochemical data used for correlation analysis between geochemistry and taxonomic composition.

**Data 3.** Relative abundances of the OTUs composed with the co-occurrence network.
